# OncomiR-10b hijacks the small molecule inhibitor linifanib in human cancers

**DOI:** 10.1038/s41598-018-30989-3

**Published:** 2018-08-30

**Authors:** Paloma del C. Monroig-Bosque, Maitri Y. Shah, Xiao Fu, Enrique Fuentes-Mattei, Hui Ling, Cristina Ivan, Nazila Nouraee, Beibei Huang, Lu Chen, Valentina Pileczki, Roxana S. Redis, Eun-Jung Jung, Xinna Zhang, Michael Lehrer, Rahul Nagvekar, Ana Carolina P. Mafra, Maria del Mar Monroig-Bosque, Alexandra Irimie, Carlos Rivera, Calin Dan Dumitru, Ioana Berindan-Neagoe, Edward P. Nikonowicz, Shuxing Zhang, George A. Calin

**Affiliations:** 1 Department of Experimental Therapeutics, The University of Texas MD Anderson Cancer Center, Houston, Texas USA; 2 Department of Medicine, University of Puerto Rico School of Medicine, San Juan, Puerto Rico; 3 Department of Medical Oncology, The First Affiliated Hospital of Xi’an Jiaotong University, Xi’an, Shaanxi China; 4 Present Address: Cell & Gene Therapy, Bioverativ Inc. A Sanofi Company, Waltham, 02451 MA USA; 5 Center for RNA Interference and Non-Coding RNAs, The University of Texas MD Anderson Cancer Center, Houston, Texas USA; 6 Center for Gene Therapy, Baylor College of Medicine, Houston, TX USA; 7 Intelligent Molecular Discovery Laboratory, Department of Experimental Therapeutics, The University of Texas MD Anderson Cancer Center, Houston, Texas USA; 8 The Research Center for Functional Genomics, Biomedicine and Translational Medicine University of Medicine and Pharmacy ‘I. Hatieganu’, Cluj-Napoca, Romania; 9 Present Address: ProQR Therapeutics N.V., 2333 CK Leiden, The Netherlands; 10 Department of Surgery, School of Medicine, Gyeongsang National University, Jin-ju, South Korea; 11 Medical and Molecular Genetics Department, Indiana University, Indianapolis, IN USA; 12 Department of Genetics, Evolution and Bioagents, Institute of Biology, University of Campinas – UNICAMP, Campinas, 13083-970 Sao Paulo Brazil; 13 Department of Biology, University of Puerto Rico, Mayaguez, Puerto Rico; 14 Department of Dental Propaedeutics and Esthetics, Faculty of Dentistry, ‘Iuliu Hatieganu’ University of Medicine and Pharmacy, Cluj-Napoca, Romania; 15 Scripps Laboratories for tRNA Synthetase Research, The Scripps Research Institute, La Jolla, California USA; 16 Translational Development and Diagnostics, Celgene Corporation, Summit, NJ USA; 17 Department of Functional Genomics and Experimental Pathology, The Oncology Institute “Prof. Dr. I Chiricuta”, Cluj-Napoca, Romania; 18 Department of Biochemistry and Cell Biology, Rice University, Houston, TX USA

## Abstract

The pervasive role of microRNAs (miRNAs) in cancer pathobiology drives the introduction of new drug development approaches such as miRNA inhibition. In order to advance miRNA-therapeutics, meticulous screening strategies addressing specific tumor targets are needed. Small molecule inhibitors represent an attractive goal for these strategies. In this study, we devised a strategy to screen for small molecule inhibitors that specifically inhibit, directly or indirectly, miR-10b (SMIRs) which is overexpressed in metastatic tumors. We found that the multi-tyrosine kinase inhibitor linifanib could significantly inhibit miR-10b and reverse its oncogenic function in breast cancer and liver cancer both *in vitro* and *in vivo*. In addition, we showed that the efficacy of linifanib to inhibit tyrosine kinases was reduced by high miR-10b levels. When the level of miR-10b is high, it can “hijack” the linifanib and reduce its kinase inhibitory effects in cancer resulting in reduced anti-tumor efficacy. In conclusion, our study describes an effective strategy to screen for small molecule inhibitors of miRNAs. We further propose that miR-10b expression levels, due to the newly described “hijacking” effect, may be used as a biomarker to select patients for linifanib treatment.

## Introduction

microRNAs (miRNAs) are small (about 21-nucleotide -long) noncoding RNAs, produced by splicing from precursor miRNA transcripts, that complementarily bind with their target messenger RNAs (mRNAs), leading to degradation or translation inhibition^1^. A plethora of reports show that specific miRNAs are aberrantly expressed in various cancers, and this dysregulation results in the activation of oncogenes or inactivation of tumor suppressor genes^2^. From a therapeutic perspective, targeting oncogenic miRNAs, which are highly overexpressed in tumors, can modulate the cancer phenotype and mediate anti-cancer mechanisms^3^. The most prevalent strategy has been to use nucleoside analogues, such as antagomiRs, chemically engineered oligonucleotides used to silence endogenous miRNAs; however, early clinical trials showed that their delivery can be ineffective or they can be outright toxic^4^. Thus, the discovery of novel therapeutic agents that could directly inhibit oncogenic miRNAs would open a new avenue of cancer opportunities to explore.

miR-10b was one of the first oncogenic miRNAs reported to play an important role in promoting tumor metastasis^5^. High miR-10b expression is associated with metastasis in several human cancers, such as pancreatic cancer^6,7^, glioblastoma^8–11^, bladder cancer^12^, and liver cancer^13^. In breast cancer, miR-10b is overexpressed in patients with metastasis^14^, especially in lymph node metastasis compared to paired primary tumors^15^. In addition, miR-10b overexpression has also been shown to promote metastasis in xenograft models of breast cancer^5^. miR-10b induces its oncogenic activity primarily through direct interaction with downstream targets, including HOXD10, NF1, KLF4, and PTEN^12,16^. Inhibiting miR-10b expression in breast cancer models can effectively suppress cancer proliferation, migration and invasion *in vitro* as well as *in vivo*^5^. In addition, combining miR-10b inhibitors with low-dose doxorubicin could achieve complete regression of metastasis in breast cancer xenograft model^17^. Studies have reported that miR-10b is also secreted by metastatic breast cancer cells via exosomes and can induce invasiveness in nonmalignant mammary epithelial cells^18^. A large-scale meta-analysis including 36 studies on 14 types of cancer found that high expression of miR-10b is significantly associated with cancer risk, and is predictive of poor patient outcome^19^. Thus, miR-10b could be a viable therapeutic target for the management of multiple types of cancer, both as a single agent and in combinatorial therapies.

In this study, we adopted a targeted approach to systematically identify specific small molecule inhibitors of miR-10b (SMIRs-10b). To this end, we tested small molecule inhibitors that have undergone first-in-human clinical trials or are already FDA approved (for any cancers and non-cancerous conditions) for the potential to target specific RNA motifs and inhibit the oncogenic function of miR-10b. Since these molecules have already undergone extensive pharmacokinetic and pharmacodynamics analysis, we hypothesized that if proven to be effective miR-10b inhibitors, their applicability in the clinic would be expanded and would greatly accelerate the lengthy process of drug discovery.

## Results

### Identification of SMIRs-10b

**Figure 1:**
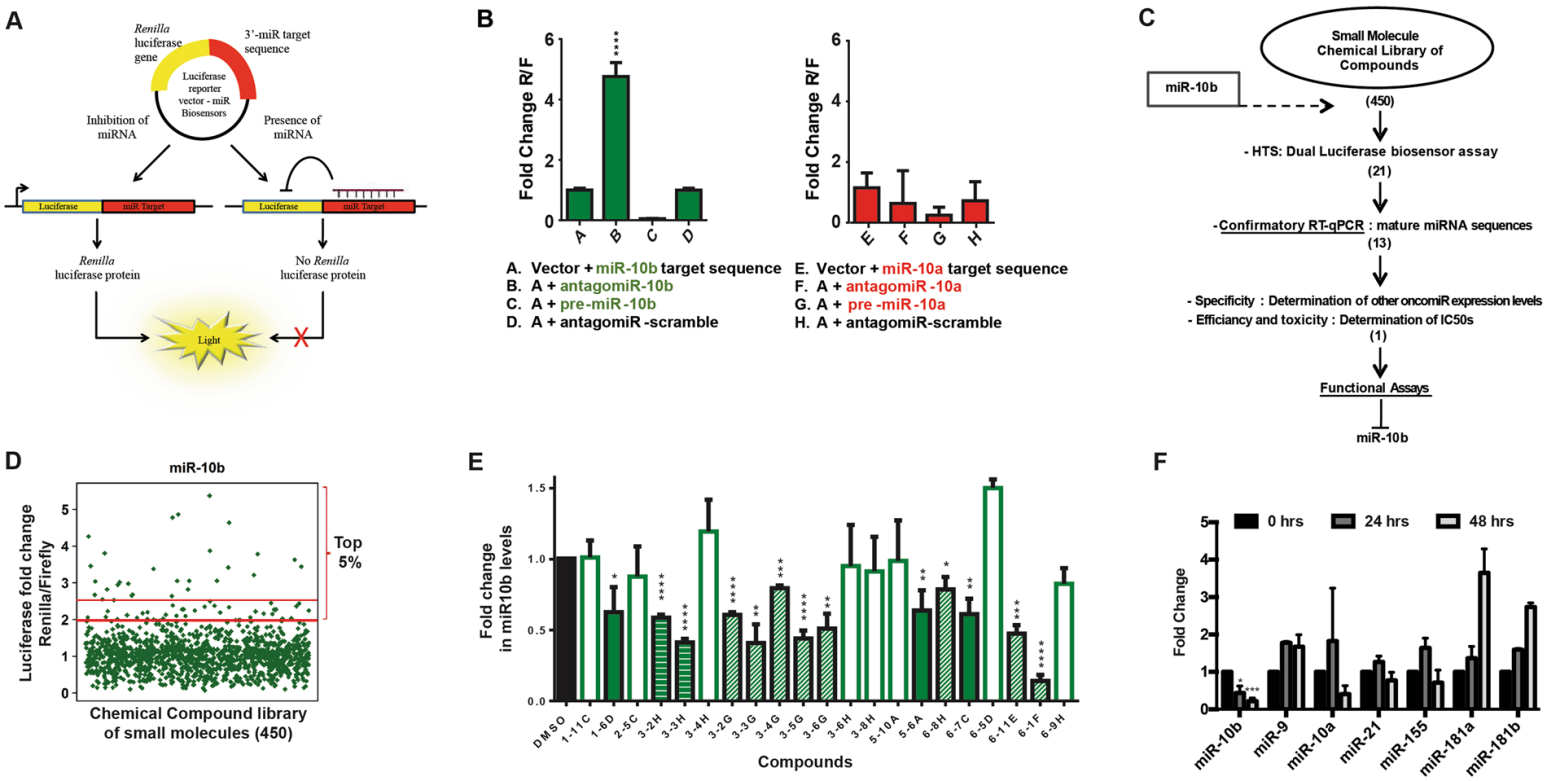
A screening system to identify potential SMIRs-10b in breast cancer. (**A)** psiCHECK-2 vector was used to clone the miR-10b target sequences downstream of a Renilla luciferase (Rluc) gene. **(B)** Sensitivity of the reporter vector construct was tested by transfecting the vectors into MCF7 cells, and introducing antagomiRs or precursor sequences for miR-10b. Luciferase expression was measured at 48 hrs after transfection. The miRNA most similar and closest to miR-10b was used as the negative control. **(C)** Schematic summarizing the steps used to define small molecule inhibitors targeting miR-10b. The number of compounds prioritized, and moved along subsequent screening steps are shown in parenthesis. **(D)** Screening results of the acquired library revealed potential SMIR-10b candidates. A total of 21 compounds from the top 5 percent with >2.5-fold luciferase expression were determined to be positive hits, and selected for further confirmatory assays. **(E)** RT-qPCR was used as a confirmatory assay that could increase the sensitivity of the screening results. We evaluated miR-10b inhibition in MCF7 cells at the same time point as the luciferase assay (48 hrs). Compounds confirmed to be positive hits in the RT-qPCR were considered “true positives”. (**F**) RT-qPCR analysis was performed to identify small molecules that targeted only miR-10b. Data for linifanib (the only miR-10b-specific small molecule) is presented. Error bars represent S.D. *represents *P* < 0.05, **represents *P* < 0.01, ***represents *P* < 0.001, and ****represents *P* < 0.0001.

To identify miR-10b inhibitors, we designed a luciferase reporter system with complementary oligonucleotides for miR-10b.This luciferase reporter would repress Renilla luciferase signal in the presence of a functional mature miR-10b. However, in the presence of a small-molecule that can inhibit mature miR-10b expression or affect its downstream function, the luciferase expression is restored again. Similar luciferase reporter systems have been used by others to identify miRNA inhibitors^20,21^. A schematic representation of the reporter construct is illustrated in Fig. 1A. We tested the sensitivity of the reporter by transfecting the vector into MCF7 breast cancer cells. Cells were also transfected with 100 nM antagomiR-10b (anti-miR-10b), or with 100 nM precursor sequences designed to mimic the effects of the miRNA (pre-miR-10b) for 48 hrs. As controls, the cells were either transfected with vector alone or with vector plus 100 nM of sequence-scrambled single stranded oligonucleotides. As shown in Fig. 1B (left panel), luciferase expression increased significantly in the presence of antagomiR-10b, and decreased significantly in the presence of pre-miR-10b. We next tested the specificity of the reporter system using antagomiR-10a and pre-miR-10a (a miRNA within the same family of miR-10b that has only one nucleotide different in the mature sequence). No significant changes were observed, suggesting adequate specificity for miR-10b (Fig. 1B, right panel). This assay was used as the initial step of our screening strategy (Fig. 1C).

We screened a library of 450 small molecules (Selleck Chemicals) for potential miR-10b inhibitors (Fig. 1D). The top 5 percent of compounds, with at least 2.5-fold luciferase overexpression (n = 21) were selected for further testing. These “positive” hits were next validated using real time-quantitative polymerase chain reaction (RT-qPCR). MCF7 cells were treated with these compounds and miR-10b expression level was detected after 48 hrs. Thirteen compounds were confirmed active (Fig. 1E). To determine possible off-target effects and eliminate compounds that could target multiple miRNAs, we tested the effect of these compounds on six clinically relevant oncogenic miRNAs (miR-9, −10a, −21, −155, −181a, and −181b)^3^ (Fig. 1F). We identified one compound, 5-6A-Linifanib (ABT-869), that was specific to miR-10b and selected it for further studies. Linifanib is a small molecule inhibitor of vascular endothelial growth factor (VEGF) and platelet derived growth factor (PDGF) families of receptor tyrosine kinases used for oral treatment of multiple advanced cancers^22^, including breast cancer^23^, colorectal cancer (CRC)^24^, non-small cell lung cancer (NSCLC)^25^, renal cell carcinoma (RCC)^26^ and hepatocellular carcinoma (HCC)^27,28^.

### Linifanib inhibits miR-10b maturation

**Figure 2:**
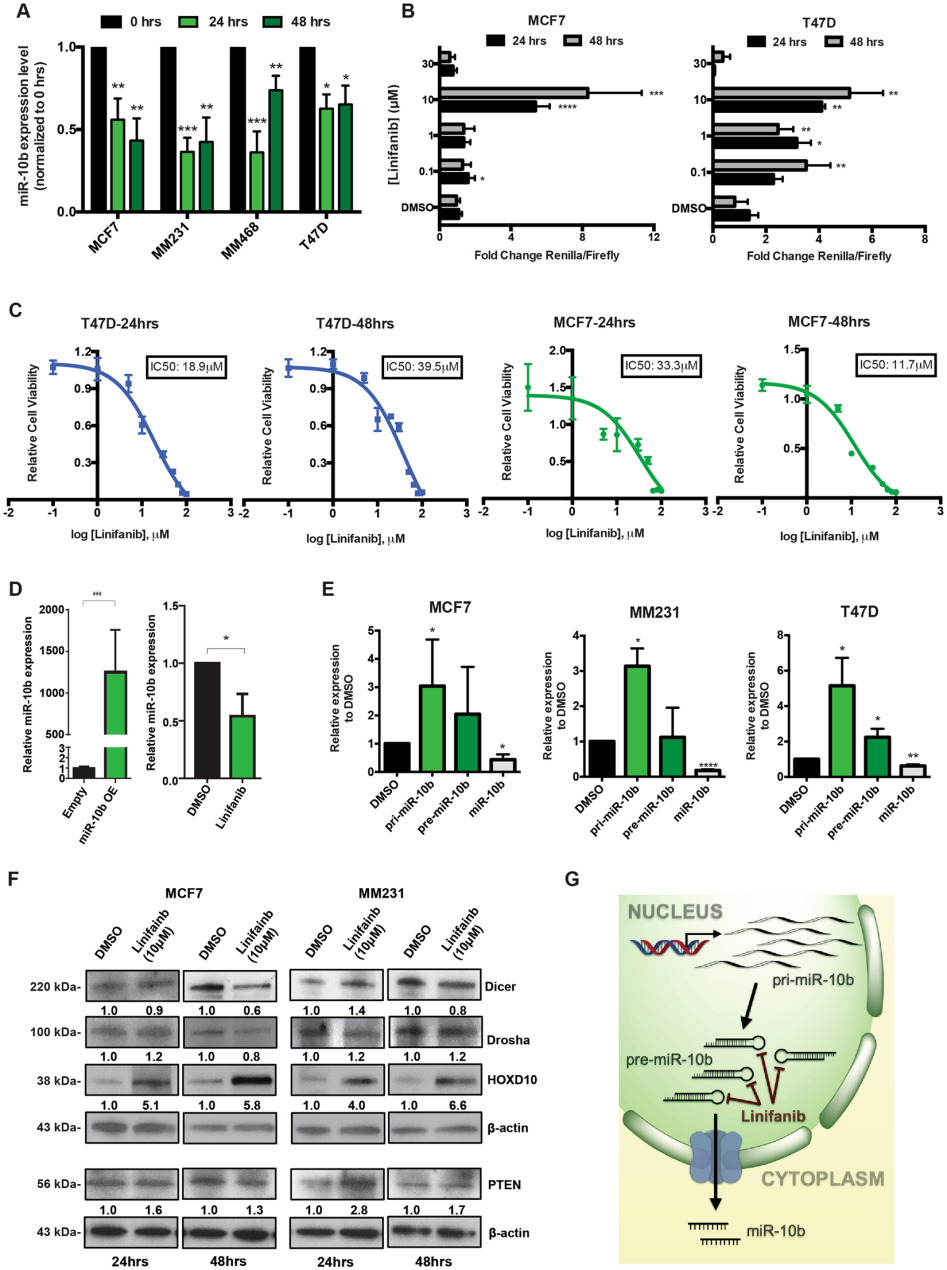
Linifanib inhibits miR-10b expression *in vitro* in breast cancer cell line models. (**A)** Treatment with linifanib (10 µM) specifically decreases miR-10b expression levels in a panel of four breast cancer cell lines. **(B)** Dosage dependent assay was done in MCF7 and T47D cells, with the luciferase-based reporter construct after 24 and 48 hrs of treatment. **(C)** The IC50s of the compound at 10 µM where calculated via MTT assay after 24 (left panel) and 48 hrs (right panel) in T47D and MCF7 cell lines. The calculated IC50s are shown in boxes. **(D)** RT-qPCR analysis to confirm miR-10b overexpression in MCF7 clones compared to the empty vectors (left panel). miR-10b expression levels were determined in these MCF7 overexpressing clones by RT-qPCR after treatment with 10 μM linifanib. **(E)** RT-qPCR analysis to determine expression levels of primary-miR-10b (pri-miR-10b), precursor miR-10b (pre-miR-10b) and mature miR-10b after treatment with 10 μM linifanib for 24 hrs in breast cancer cell lines. **(F)** Western blots showing expression levels of Dicer, Drosha, HOXD10 and PTEN after treatment with 10 μM linifanib for 24 and 48 hrs in breast cancer cell lines. **(G)** Potential mechanism of action of linifanib: interaction with precursor miR-10b and reduction in the production of mature miR-10b with accumulation of primary and precursors transcripts of miR-10b. Error bars represent S.D. *represents *P* < 0.05, **represents *P* < 0.01, and ***represents *P* < 0.001.

We then validated linifanib’s anti-miR-10b activity in additional breast cancer cell lines (Fig. 2A). Linifanib inhibited miR-10b expression in MCF7 (derived from an invasive ductal breast carcinoma), MDA-MB-231 (MM231), MDA-MB-468 (MM468) and T47D, all three derived from metastatic pleural effusions of mammary adenocarcinomas (for details see Supplementary Table 1). We next performed dose-dependent luciferase assays to determine the potency of miR-10b inhibition. Using the previously cloned psiCHECK2 vector, we tested four different concentrations (ranging from 0.1–30 µM) at two different time-points (24 and 48 hrs). Linifanib showed maximum miR-10b inhibition at 10 µM concentration in both MCF7 and T47D cell lines (Fig. 2B). Linifanib’s IC50 (the concentration required for 50% inhibition *in vitro*) was calculated using the colorimetric MTT assay after 24 and 48 hrs of treatment. In MCF7 cells, linifanib’s IC50 was 33.3 µM and 11.7 µM after 24 and 48 hrs respectively. While in T47D, linifanib’s IC50 was 18.9 µM and 39.5 µM after 24 and 48 hrs respectively (Fig. 2C). Next, we tested the sensitivity of linifanib’s anti-miR-10b activity by treating miR-10b-overexpressing MCF7 clones with linifanib. Once again, we found that linifanib was able to reduce the mature miR-10b expression by almost 50%, even when the miRNA was overexpressed >1000X over normal levels (Fig. 2D). Thus, we selected 10 µM concentration to perform additional functional and mechanistic analysis, since linifanib showed maximum miR-10b inhibition at this concentration.

Next, we wanted to determine how linifanib inhibited miR-10b. We hypothesized that it could function by either inhibiting miR-10b’s biogenesis or by inhibiting its downstream function. For this purpose, we analyzed the expression levels of primary miR-10b (pri-miR-10b) and precursors of miR-10b (pre-miR-10b), as well as some confirmed downstream target genes after treatment with 10 µM linifanib. We identified that pri-miR-10b and pre-miR-10b expression levels significantly increased, while mature miR-10b levels decreased in all three breast cancer cell lines (Fig. 2E). The data strongly implied that linifanib inhibited endogenous miR-10b processing from primary and precursor to the mature form, and the “slowdown” of miR-10b maturation led to the accumulation of pri-miR-10b and pre-miR-10b. Under the same conditions, we tested for the levels of the two most important proteins required for miRNA biogenesis and maturation: the endoribonuclease Dicer and the ribonuclease III superfamily member Drosha. Linifanib treatment did not significantly modify the levels of any of these enzymes, suggesting that the general miRNA biogenesis machinery was not significantly altered (Fig. 2F). In addition, western blot analysis showed that linifanib successfully inhibited miR-10b downstream targets PTEN and HOXD10 (Fig. 2F). Thus, our data suggested that linifanib specifically inhibits biogenesis of mature miR-10b, and thus downregulates its downstream pathway.

### Linifanib inhibits miR-10b’s oncogenic function *in vitro*

**Figure 3:**
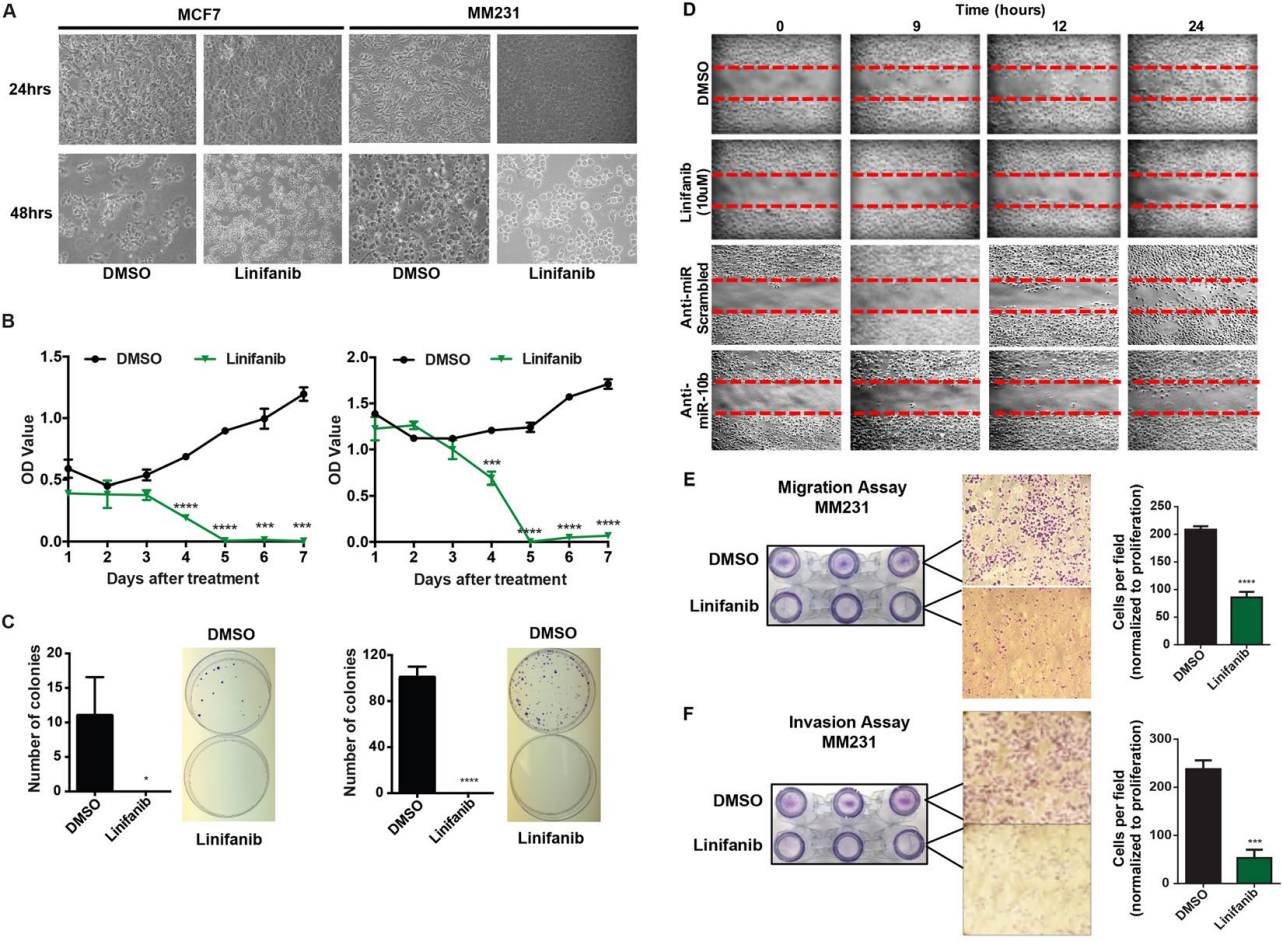
Linifanib inhibits miR-10b’s oncogenic function *in vitro*. (**A)** Morphological analysis of MCF7 and MM231 cell lines after 24 and 48 hrs of treatment with 10 μM linifanib. **(B)** BrdUproliferation assay to determine cell proliferation after treatment with 10 μM linifanib in MCF7 (left panel) and MM231 (right panel) cell lines. **(C)** Colony formation analysis in MCF7 (left panel) and MM231 (right panel) cell lines after treatment with 10 μM linifanib after 14 days. **(D)** Scratch assay to determine migration capacity of MM231 cells after treatment with 10 μM linifanib. Migration **(E)** and invasion **(F)** analysis in MM231 cells after treatment with 10 μM linifanib for 24 hrs. Error bars represent S.D. *represents *P* < 0.05, **represents *P* < 0.01, ***represents *P* < 0.001, and ****represents *P* < 0.0001.

We next evaluated the *in vitro* antitumor efficacy of linifanib using breast cancer cell lines. Treatment of MCF7 and MM231 cell lines with 10 µM linifanib induced significant alteration in the morphology of the cells after 24 hrs (Fig. 3A). BrdU Cell Proliferation Assay demonstrated that 10 µM linifanib induced significant cell death at 3 days after treatment, and almost completely eliminated all cells following day 4 (Fig. 3B). Additionally, treatment with 10 µM linifanib also significantly inhibited the colony formation capability of MCF7 and MM231 cell lines (Fig. 3C). Thus, our data demonstrated that linifanib can significantly inhibit proliferation of breast cancer cell lines.

Since miR-10b has been known to induce migration and invasion, we analyzed the migration and invasion of MM231 cell line (which has an endogenous high level of miR-10b), after treatment with 10 µM linifanib. A wound healing assay revealed that linifanib inhibited migration of MM231 cells (Fig. 3D). The results were comparable to treatment with antagomiR-10b (Fig. 3D). We then performed a matrigel-based migration and invasion assay in MM231 cells after treatment with 10 µM linifanib and normalized the results for proliferation. We found that even after normalization, treatment with linifanib had significantly decreased migration and invasion by 50% and 75%, respectively (Fig. 3E,F). In conclusion, our data demonstrated that linifanib could significantly inhibit proliferation, migration and invasion *in vitro*.

### Linifanib and anti-miR-10b treatments have similar anti-cancer effects *in vivo*

**Figure 4:**
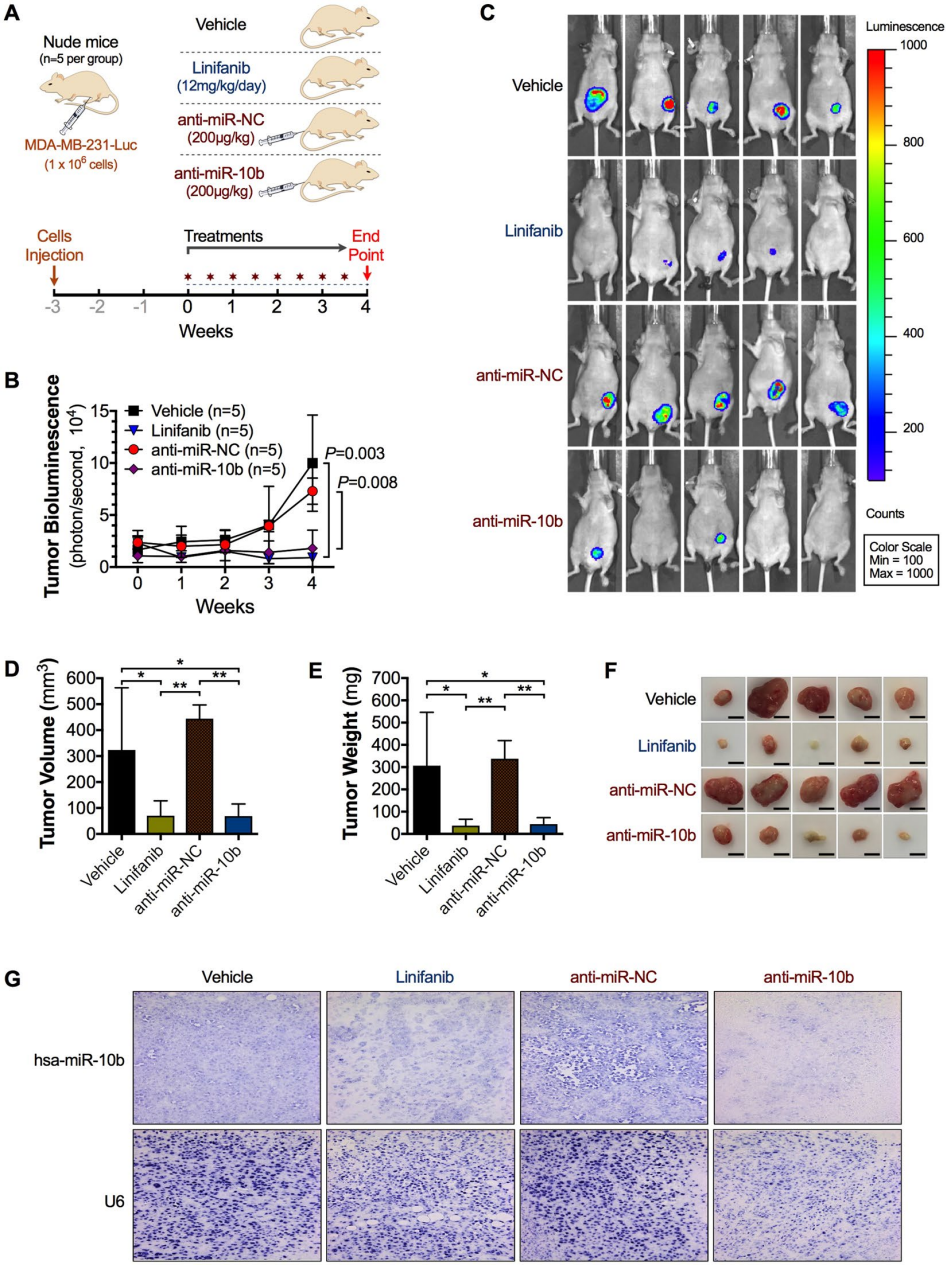
Linifanib inhibits miR-10b’s oncogenic function *in vivo*. (**A**) Schematic representation of the *in vivo* experiment. **(B)** Line chart of the integrated tumor bioluminescence (n = 5 mice per group) at different weeks after treatment. **(C)** Representative *in vivo* bioluminescent imaging of tumors of allografted MM231-FG12-Luc cells performed 4 weeks after treatments (n = 5 mice per group). **(D)** Bar graph illustrating the mean tumor volumes from the orthotopic allografted tumors harvested from mice (n = 5 mice per group). **(E)** Bar graph illustrating the mean tumor weights from the harvested orthotopic allografted tumors (n = 5 mice per group). **(F)** Representative images of harvested orthotopic allografted tumors (scale bars represent 5 mm). **(G)** Representative images of *In Situ* Hybridization of fixed tumor tissues for miR-10b and U6 as internal control. Statistical significance was calculated by one-way analysis of variance. Error bars represent S.D. *Represents P < 0.05, **represents P < 0.01, and ***represents P < 0.001.

To compare the *in vivo* activity of linifanib and anti-miR-10b treatment in breast cancer, we established an orthotopic breast cancer mouse model by mammary fat pad injection of luciferase-expressing MM231 (MM231-FG12-Luc) cells. Three weeks after injection, the mice were randomized in four treatment groups as described in Fig. 4A. Linifanib (12 mg/kg/day) was administered through oral gavage every day, while liposomal nanoparticles containing anti-miR-10b or scrambled control (0.2 mg/kg) were administered intravenously (IV) every 72 hrs. Mice treated with linifanib showed a significant decrease in total tumor bioluminescence, tumor volume and tumor weight when compared with mice treated with vehicle or scrambled control (Fig. 4B-F). We further observed that linifanib displayed antitumor efficacy similar to anti-miR-10b *in vivo*, suggesting that it was equally efficient at inhibiting miR-10b *in vivo*, as was expected based on the *in vitro* data. *In situ* hybridization on tumor tissue confirmed that miR-10b expression levels were significantly reduced in mice treated with linifanib or with anti-miR-10b when compared to vehicle or scrambled treated mice, respectively (Fig. 4G). Thus, our data thoroughly indicated that linifanib induces strong antitumor activity by inhibiting miR-10b *in vivo*, this in addition to its inhibitory effects on tyrosine kinases that has already been shown^22,29^.

### Linifanib inhibits miR-10b in other cancers

**Figure 5:**
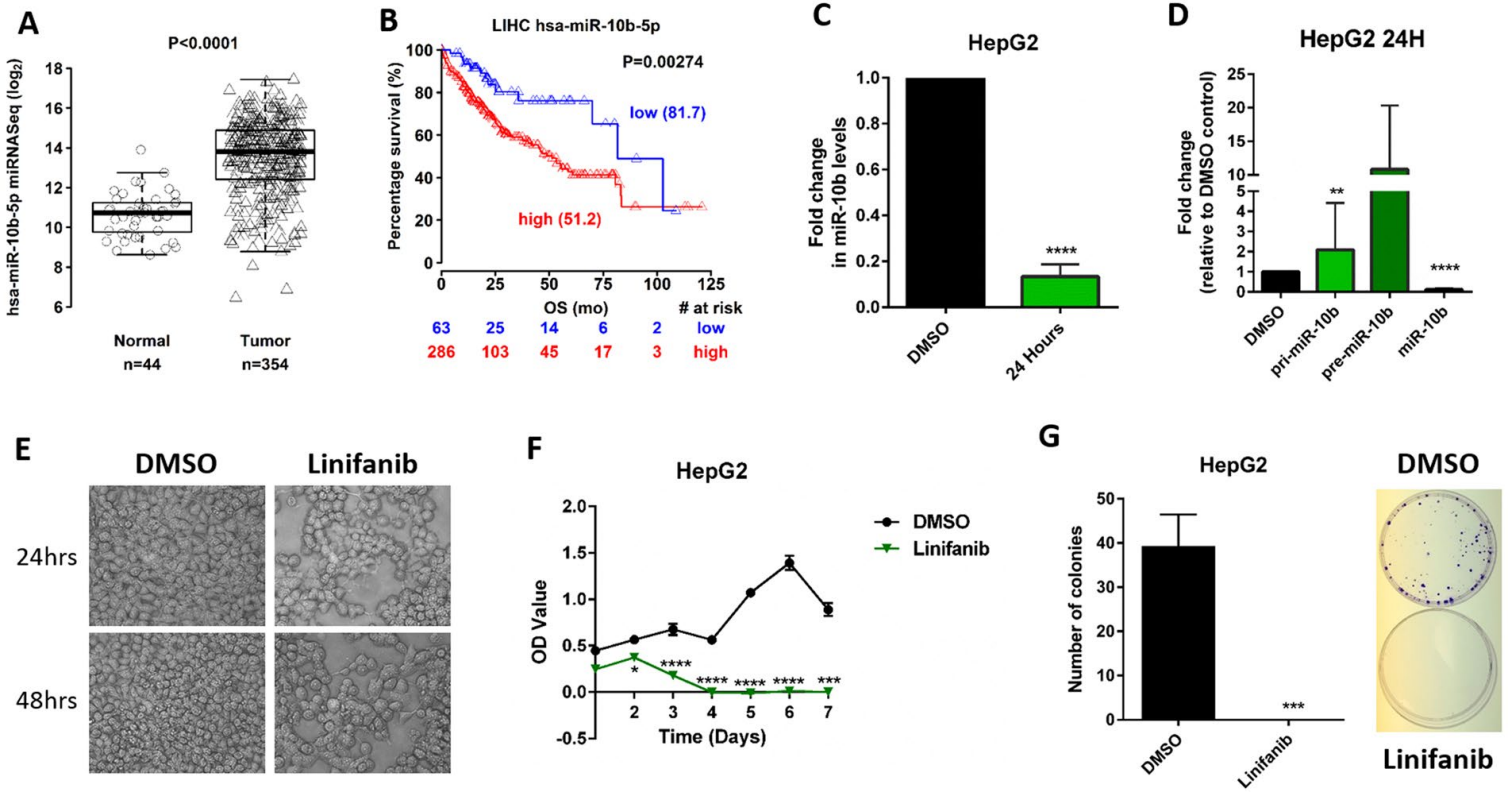
Linifanib inhibits miR-10b in HCC cells. (**A**) miR-10b is significantly upregulated in the HCC tissues compared to normal liver tissues. (**B**) The median survival rates for HCC patients with high- and low- expression of miR-10b from TCGA dataset. (**C)** Treatment with 10 µM linifanib significantly decreases miR-10b expression levels in HepG2 cell line. **(D**) RT-qPCR analysis to determine expression levels of pri-miR-10b, pre-miR-10b and mature miR-10b after treatment with 10 μM linifanib for 24  hrs in HepG2 cells. The expression of pri-miR-10b, pre-miR-10b and miR-10b in HepG2 cells after treatment with 10 μM DMSO for 24 hrs were set as control. (**E)** Morphological analysis of HepG2 cells after 24 (upper panel) and 48 (lower panel)  hrs of treatment with 10 μM linifanib or DMSO. (**F**) BrdU proliferation assay to determine cell proliferation after treatment with 10 μM linifanib or DMSO in HepG2 cells. (**G**) Colony formation analysis in HepG2 cells after treatment with 10 μM linifanib or DMSO after two weeks. Error bars represent S.D. *represents *P* < 0.05, **represents *P* < 0.01, ***represents *P* < 0.001, and ****represents *P* < 0.0001.

We further evaluated if linifanib could target miR-10b in additional cell lines from other cancer types for which this miRNA has been shown to be overexpressed and to contribute to tumorigenesis. We focused on HCC, as this is a deadly type of cancer and linifanib has been extensively tested with positive results in HCC^28^. By analyzing the The Cancer Genome Atlas (TCGA) dataset, we identified that miR-10b is significantly upregulated in the HCC tissues compared to normal liver tissues (*P* < 0.0001, Fig. 5A). We also found that a high level of miR-10b leads to poor prognosis in HCC patients (the median survival rates for HCC patients with high- and low- expression of miR-10b are 51.2 and 81.7 months respectively, *P* = 0.00274, Fig. 5B). By using HepG2 cells we demonstrated that linifanib reduces miR-10b levels (Fig. 5C). Linifanib also inhibits maturation of miR-10b in HepG2 cells, resulting in accumulation of precursor forms of the miRNA: pri-miR-10b and pre-miR-10b (Fig. 5D). Finally, linifanib successfully inhibits proliferation and colony formation in HepG2 cells (Fig. 5E–G). Thus, we concluded that linifanib induces antitumor activity in multiple cancer models by effectively downregulating miR-10b and altering the downstream pathways regulated by oncogenic miR-10b.

### High level of miR-10b hijacks linifanib and prevents it from inhibiting its kinase targets in cancers

**Figure 6:**
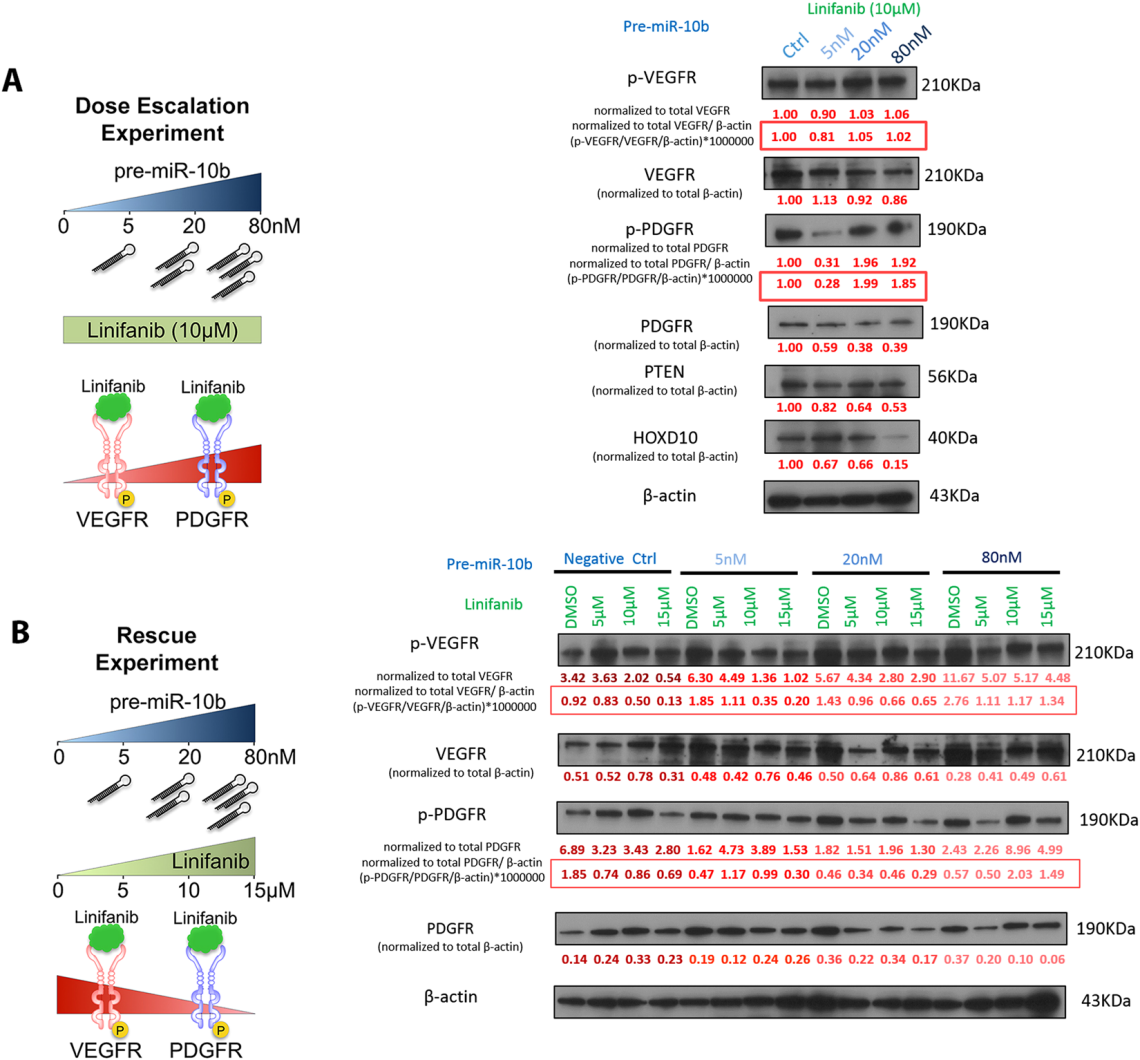
High level of miR-10b “hijacks” linifanib and prevents it from inhibiting its kinase targets in cancers. (**A**) Schematic representation of dose-dependent experiments (left panel) and western blots (right panel) showing the expression of p-VEGFR and p-PDGFR increased, but the expression of total VEGFR and PDGFR as well as HOXD10 and PTEN decreased after transfection with increasing doses of pre-miR-10b (0, 5, 20, 80 nM) and treatment of 10 μM linifanib for 24 hrs. On the left side of the blots, proteins names and the normalization methods are presented. (**B**) Schematic representation of rescue experiments (left panel) and western blots (right panel) showing the p-VEGFR and p-PDGFR decrease mediated by increasing doses of linifanib (0, 5, 10, 15 µM) could be rescued when transferring increasing doses of pre-miR-10b (0, 5, 20, 80 nM) in HepG2 cells. On the left side of the blots, proteins names and the normalization methods are presented.

We next tested the hypothesis that the miR-10b interaction with linifanib, direct or indirect, reduces the drug inhibition of VEGF and PDGF receptors. We termed this the “hijacking” effect of miR-10b. To test this, we performed two types of experiments using HepG2 cell line (we chose this cell line because it has higher expression of VEGFR and PDGFR compared to the breast cancer cell lines we investigated). First, we ran a dose-escalation experiment in which we transfected HepG2 with increasing amount of pre-miR-10b (0, 5, 20, 80 nM) and treated it with an equal dose of linifanib (10 µM) for 24 hrs (Fig. 6A). Western blot analysis showed that the phosphorylated PDGFR (p-PDGFR), and at a lower extent p-VEGFR, increased, but total VEGFR and PDGFR decreased (Fig. 6A). On the same blots, HOXD10 and PTEN, two known targets of miR-10b also decreased, proving that miR-10b was as well interacting with its main known targets. To further confirm that miR-10b could “hijack” linifanib, we conducted rescue experiments by transferring increasing doses of pre-miR-10b (0, 5, 20, 80 nM) and then treated HepG2 with increasing doses of linifanib (0, 5, 10, 15 µM) for 24 hrs (Fig. 6B), that would minimize the “hijacking”. Western blot analysis showed that the p-VEGFR and p-PDGFR increase mediated by linifanib could be rescued when treating with increasing doses of pre-miR-10b, as proved by comparing the different doses of linifanib and pre-miR-10b versus DMSO control (Fig. 6B). Our results confirmed that high levels of miR-10b can “hijack” linifanib and inhibit its pharmacologic purpose of targeting and decreasing VEGFR and PDGFR.

In conclusion, our data strongly suggested that linifanib induces antitumor activity in multiple cancer models by specifically inhibiting maturation of miR-10b as well as interfering its interaction with its downstream targets.

## Discussion

Ninety percent of cancer-related mortalities are caused by metastasis. Despite significant progress achieved in detection and anticancer therapies for breast or HCC, the majority of the promising results occur only in patients treated in early stages^30^. Recurrence is frequent, and aggressive metastatic disease is incurable. MiRNA-based therapeutics offer an alternative approach to treat advanced disease, due to their ability to simultaneously target multiple effector pathways involved in a plethora of cellular processes (e.g. proliferation, differentiation, survival, etc.). These compounds are being developed either alone or in combination with current targeted therapies, with the ultimate goal of improving disease response and increasing the cure rates^2^. However, the delivery mechanisms required for oligonucleotide therapeutics have significant shortcomings, as they can be ineffective, non-specific and even toxic *in vivo*^31^.

The concept of targeting miRNAs with small molecules has been poorly explored. Gumireddy *et.al*^20^ reported the first study in the field, which by structure–activity relationship analyses identified diazobenzene as an inhibitor of miR-21 transcription and function. Shi et.al developed a screening system that yielded a small molecule that could target miR-21’s oncogenic functions *in vivo*^21^. Similarly, the small molecule enoxacin (a fluoroquinolone) was demonstrated to enhance the production of miRNAs with tumor suppressor functions *in vivo*^32^. In this study, we screened 450 small molecule inhibitors to test their ability to inhibit miR-10b. The data presented here further expands the growing field of miRNA-targeting small molecule inhibitors and identified the first small molecule inhibitor of oncogenic miR-10b.

Through luciferase-based screening and further miR-10b inhibition analysis, we identified linifanib as a specific miR-10b inhibitor that blocks miR-10b biogenesis (but not of others oncogenic miRNAs). Linifanib (ABT-869) is a potent inhibitor of VEGFR and PDGFR^22^. In nonclinical *in vivo* studies, linifanib demonstrated potent inhibition of tumor growth in xenograft models of over 15 different tumor types^29^. In preclinical studies, it was also proven to have antitumor activity as a single agent, as well as in combination with chemotherapies^24^. To date, linifanib has been shown to be clinically active in patients with an acceptable safety profile; indeed, a total of 18 clinical trials have been initiated in diseases including solid tumors such as breast cancer^23^, HCC^27,28^, CRC^24^, NSCLC^25^ and RCC^26^. Despite these encouraging data, the clinical trials with this compound were terminated early because no significant overall survival advantage was observed over other compounds from the same class (such as sorafenib)^28^. However, our study proposes that linifanib derivatives with higher affinity for miR-10b precursor could be attractive agents to target metastatic breast cancer (and others such as HCC) by inhibiting the “oncomiR-10b addiction” of these tumor types. Silencing of miR-10b markedly suppresses formation of breast cancer and HCC metastasis both *in vitro* and *in vivo*. By inhibiting miR-10b and indirectly restoring the levels of its tumor suppressor targets HOXD10 and PTEN, linifanib decreased proliferation, invasion and migration of breast cancer and HCC cells.

Strikingly, although we observed that linifanib could inhibit miR-10b, its anti-cancer effects were simultaneously reduced when miR-10b levels were high since high miR-10b inhibited linifanib’s interaction with VEGFR and PDGFR. We argue that high miR-10b could potentially “hijack” linifanib, resulting in less available linifanib to interact with and inhibit its intended kinase targets (VEGFR and PDGFR). In other words miR-10b, lowers its clinical efficacy. Such an unexpected effect implies that miR-10b could be used as a biomarker to stratify cancer patients for treatment with linifanib: low dosage of linifanib could be used to treat patients with low levels of miR-10b, thus reducing toxicity and side effect associated with linifanib. On the other hand, for patients with high miR-10b, linifanib is not an ideal treatment option since miR-10b “hijacking” will prevent its tumor suppressor function, resulting in higher dosage and more off-target toxicities. In these patients, sorafenib could be of more benefit or the linifanib derivatives with higher affinity for miR-10b precursor. Furthermore, our study raises the concern that some SMIRs developed over the years (with thorough work and huge financial investments) might have been discontinued not because of true poor performance, but because of an unknown underlying molecular mechanism involving miRNA “hijacking”. By profiling specific interactions, these underlying factors could allow the discovery of potential therapeutic alternatives for personalized medicine.

Notably, the majority of the cancer types involved in clinical trials on linifanib (including breast cancer) have been shown to worsen with increased miR-10b expression. For example, miR-10b is highly expressed in metastatic HCC inducing invasion and migration; and patients with higher miR-10b expression have significantly poorer overall survival^13,33^. Similarly, high miR-10b expression was found to be significantly associated with a high incidence of lymphatic invasion and poor prognosis in CRC patients and has been shown to confer resistance to chemotherapeutic agents *in vitro*^34^. In NSCLC, miR-10b expression levels were significantly positively correlated with TNM stage and regional lymph node involvement, and patients with higher levels of miR-10b have significantly poorer survival than those with lower expression of this miRNA^35,36^. In this regard, we believe that inhibition of miR-10b (along with its known targets) by linifanib derivatives could further contribute to its therapeutic efficacy in preclinical models and clinical trials of different cancers that have been proven to be oncomiR-10b addictive, such as pancreatic cancer and glioblastoma^37–39^. We also favor that in combination with other agents, linifanib or a derivative could increase the positive outcomes of cancer patients.

In our screening, we observed and validated a new mechanistic role for linifanib via the post-transcriptional inhibition of mature miR-10b, which has been found to be overexpressed in several cancer types. In both breast cancer and HCC models, we observed that linifanib decreased the levels of miR-10b with accompanied accumulation of pre-miR-10b. The sensitivity of the inhibition also was consistent among different cell line models. The specific molecular mechanism is yet to be elucidated with ongoing investigations. However, one possible mechanism that could give rise to the selective inhibition of miR-10b by linifanib is a direct interaction with pre-miR-10b. However, the current identification of direct miRNA-small molecule interactions is hindered by technological limitations due to the large size and low stability of pre-miR-10b and the cellular environmental conditions in which miR-10b exists. We used multiple techniques including nuclear magnetic resonance (NMR), isothermal titration calorimetry (ITC), and surface plasmon resonance (SPR) to probe for linifanib-miR-10b specificity *in vitro*, but the results of these experiments were not robustly reproducible; this represents a big issue in the field that should be addressed by developing novel technologies to accurately prove small molecules directly interact with RNA.

In conclusion, our study describes an effective strategy to screen for SMIRs. Our results lead to the identification of linifanib as a SMIR-10b compound. We further propose that miR-10b expression levels, due to the newly described “hijacking” effect, may be used as a biomarker to select patients for linifanib treatment.

## Methods

### Cell Culture

Cell lines were obtained from the American Type Culture Collection and grown as suggested by the supplier. Experiments were performed using MCF7, MM231, MM468, and HepG2 cell lines cultured in DMEM supplemented with 10% Fetal Bovine Serum and maintained at 37 °C in a humidified atmosphere of 5% CO2. miR-10b-overexpressing MCF7 clones were generated by miR-10b-pcDNA3 plasmid, and the stable clones were selected in media containing G418. MDA-MB-231-FG12-Luc (MM231-FG12-Luc) cells for *in vivo* imaging was generated by lentiviral infection with FG-12 vector to express both green fluorescent protein and luciferase as previously described^40^.MM231-FG12-Luc cells were selected by sorting for green fluorescent protein expression using FACS Aria II (BD Bioscience).

### Reporter Plasmid Construction

We acquired the Psi-CHECK 2 luciferase reporter vector (Promega). We then designed and obtained complementary oligonucleotides (Thermo Fisher Scientific) for hsa-miR-10b based on the mature miRNA sequence. In the design of the miRNA target sequence, we added several base pairs to have two different restriction sites recognizable by the enzyme SgfI at one side, and PmeI on the other (present in the vector as well). In addition, the insert was designed with a restriction site for SacI enzyme in order to have an additional site at which to digest the vector when monitoring colonies for the presence/absence of the insert. The designed oligonucleotides were annealed as follows. Complementary strands were mixed at 1:1 molar ratio in a micro centrifuge tube. The mixture was diluted to a final concentration of 1 pmol/µl with a buffer solution of 10 mM Tris, 1 mM EDTA, and 50 mM NaCl (pH 8.0). Mixes were incubated at 95 °C for 5 minutes, and then the heat was reduced gradually for 70 minutes, until reaching 4 °C. The annealed oligonucleotides were digested along with the Psi-CHECK2 vector with SgfI and PmeI and ligated at the 3′-UTR (downstream) of the Renilla luciferase reporter gene in the psiCHECK-2vector. Insert sequences were further confirmed in two ways: enzymatic digestion and sequencing.

### Molecular-Luciferase-Based Small Molecule Screening

A library screening of inhibitors lyophilized and dissolved in DMSO was purchased (Selleck Chemicals). MCF7 cells were seeded 24 hrs prior to transfection in 96 well plates (2.0 × 10^4^ cells/well). Sensitivity Assay: To test the sensitivity of the biosensor, we used antagomiRs and pre-miRs against miR-10b at a final concentration of 100 nM. The assay was done in MCF7 cells at approximately 60% confluence using Lipofectamine in Opti-Mem media with the final vector concentration of 250 ng/mL. All transfections were performed in triplicates for statistical analysis. The cells were incubated at 37 °C for 6 hrs, followed by replacement of the transfected media with fresh media. After 48 hrs incubation, the media was removed, and cells were lysed and assayed with a Dual Luciferase Assay Kit (Promega) using a Vi-Tech luminometer. The ratio of Renilla to Firefly luciferase expression was calculated for each of the triplicates. Screening: To test the small molecules effects the experimental conditions were kept as previously described, and the drugs were added to a final concentration of 10 µM.

### RNA extraction

The media was removed, cells were washed with PBS, and RNA was isolated using Trizol Reagent (Invitrogen), according to manufacturer’s instructions. RNA quantity was assessed with NanoDrop ND-1000 (Thermo Fisher Scientific) and the integrity was analyzed by gel electrophoresis.

### Quantitative Real Time PCR Analysis

The primary screening hits were selected to confirm their potential as SMIRs. Their inhibition was tested in MCF7 cells, a metastatic cell line (MM231) and an HCC cell line (HepG2) through RT-qPCR. Cells were seeded in 12-well plates 24 hrs before treatments at a confluency of 50–60%. They were treated at a concentration of 10 µM linifanib or DMSO (as control), and RNA was collected after 24 and 48 hrs. MiRNA expression was tested using TaqMan microRNA assay (Applied Biosystems). The cDNA was synthesized using TaqMan Reverse Transcription Reagents kit (Applied Biosystems) and employed for RT-qPCR analysis together with TaqMan probes and SsoFastSupermix (Bio-rad). Primers and probes were purchased for hsa-miR-10b, hsa-pri-miR-10b and U6 snRNA that was used as an internal control.

To detect the levels of the precursor sequences, cDNA was synthesized using the SuperScript III cDNA kit (Invitrogen), and diluted cDNA was used for RT-qPCR analysis using iQ SYBR Green Supermix (Bio-Rad) with primers designed by us (Supplemental Table 2). Experiments were performed in triplicates; treated samples were compared to the DMSO-treated controls and normalized to the internal control. Relative expression levels were calculated using the 2^−ΔΔCt^method.

### Dose-Dependent Cell-based assays

The luciferase-reporter (PsiCHECK2) vector construct was used to test the half maximal inhibitory concentration (IC50) at which each SMIRs induced a response halfway between the baseline and maximum after a specified exposure time. Cells were plated at a 50–60% confluence 24 hrs before transfection. Transfection was performed as initially done for the primary screen, and effectiveness of SMIRs was tested at two separate time-points 24 and 48 hrs. A total of five different concentrations were tested in serial dilutions and the IC50 was determined for each compound using MCF7 cells.

### Lentivector-based miRNA Precursor Constructs

We designed primers for the precursor sequences of each miRNA (OligoPerfectTM Designer) including BamH1 and EcoR1/Not1 restriction sites and cloned them in the pCDH-CMV-MCS-EF1-copGFP vector. For the lentiviral production, 293FT cells were grown in DMEM supplemented with 10% FBS and passed when they were 80% confluent. Cells were transfected in 10 cm dishes when 60% confluent with 8.0 µg of transfer vector (pMirna), 5.2 µg of the packaging vector (CDH-CMV MCS-EF1-copGFP) and 2.8 µg of the envelope vector (pMD.G). After 48 hrs, the supernatant containing the virus was collected and filtered (0.45 µm). MCF7 cells were then infected with the viral supernatant containing either the empty vector, or vector containing the target sequence for miR-10b along with Polybrene/Sequabrene antibiotic, at a final concentration of 8 µg/mL. The plate was then centrifuged 1500–1800g for 90 minutes, incubated at 37 °C for 30 minutes and the culture media replaced with fresh one. Transfection efficiency was monitored through GFP, as well as confirmed by RT-qPCR.

### Cell Viability

Cell viability and IC50 were determined by the 3-(4, 5-dimethyl-2-thiazolyl)-2, 5-diphenyl-2H-tetrazolium bromide (MTT assay). Briefly, 10,000 MCF7 and T47D cells were plated the evening prior to treatment in 96 well microculture plates. After cells became adherent, five different doses of the drugs were added to the supernatant of newly added media. After 24 and 48 hrs, the MTT reagent (Sigma) was added to each well and incubated for 3 hrs at 37 °C. The optical density (OD) was read at 570 nm on a microplate spectrophotometer and growth values (%) were calculated as (OD treated cells/OD untreated cells) × 100. The experiments were performed in quadruplicate.

### Proliferation assay

Cell proliferation was determined using a colorimetric assay were the highly water-soluble tetrazolium salt WST-8 (Dojindo Molecular Technologies), which is reduced by dehydrogenase activities in cells to give a yellow-color formazan dye, which is soluble in the tissue culture media. The amount of the formazan dye (generated by the activities of dehydrogenases in cells, and directly proportional to the number of living cells) was measured after 3 hrs of incubation. The OD was read at 450 nm on a microplate spectrophotometer and growth values (%) were calculated as (OD treated cells/OD untreated cells) × 100. The experiments were performed in triplicate. Cell viability was also tested by bromodeoxyuridine (BrdU) test according to the instructions. 2000 MM231, MCF7 and HepG2 cells were seeded to 96-well plate. After 24 hrs, 10 μM DMSO or linifanib was added to each well. The optical density (OD) was read at 450/550 nm on a microplate spectrophotometer, after one to seven days of the treatment. The experiments were performed in triplicate.

### Colony formation assay

500 MM231, MCF7, and HepG2 cells were added to 6-well plate. 24 hrs later, 10 μM DMSO or linifanib was added to each well. Two weeks later, colonies were fixed with 4% paraformaldehyde, stained with crystal violet and counted using a stereomicroscope.

### *In vitro* migration and invasion assay

In the cell motility assay, 100 μL of serum-free media containing 50,000 cells (MM231) were seeded onto the insert with 8.0 µm porous membrane well either coated with gelatin (for migration assay) or Matrigel matrix containing collagen and laminin (for invasion assay), and 500 μL of media with serum was added to the bottom well. Cells were left to migrate or invade for 24 hrs. The cells that migrated or invaded to the bottom of the well were fixed, stained and counted. For each well, five different fields were counted and the average number of cells was determined (AutoCAD). The experiments were performed in triplicates. Results from both assays were normalized to proliferation.

### Western Blot

Proteins were collected from cultured cells and lysed with 10X Lysis Buffer (Cell Signaling) freshly supplemented with a complete protease inhibitor cocktail and phosphatase inhibitors (Sigma). Bradford assay was used to measure protein concentration. Proteins were separated by polyacrylamide gel (Bio-rad) electrophoresis and were transferred to a 0.2 µm nitrocellulose membrane (Bio-rad). The list of antibodies is described in Supplementary Table 3.

### *In vivo* experiments

*In vivo* orthotopic grafting experiments were performed by injecting MM231-FG12-Luc cells in 30 μL of saline solution containing 50% (v/v) of Reduce Growth Hormone Matrigel (BD Bioscience) into the left fourth mammary fat pad of female Nu/Nu mice. *In vivo* imaging of tumors was performed using a Xenogen IVIS 100 optical *in vivo* imaging system. The *in vivo* experiments were conducted in accordance with American Association for Laboratory Animal Science regulations and the approval of The University of Texas MD Anderson Cancer Center Institutional Animal Care & Use Committee.

### Statistical Analysis

All statistical analyses in this study were performed in R (version 3.0.1). All tests were 2-sided and considered statistically significant at the 0.05 level. The RT-qPCR, luciferase dosage dependent analysis, proliferation, migration and invasion assays were performed in triplicates. The MTT and BrdU experiments where performed in quadruplicates. A (two-sided) t-test was applied to compare the mean between control vs. treated samples and analyses were carried out in GraphPad (Prism 6). Log-rank test was used to find the point (cut-off) with the most significant (lowest p-value) split in high vs low miRNA level groups. The Kaplan-Meier plots were generated for these cut-off (0.18). The numbers of patients at risk in low and high miR-10b groups at different time points are presented at the bottom of the graph. In brackets, we present median survival months in each group. The statistical significance was defined as a *P*-value less 0.05. All data are represented as standard deviation (S.D.) of the mean.

## Electronic supplementary material

Supplementary Dataset
