# Supplementary material for: OncomiR-10b hijacks the small molecule inhibitor linifanib in human cancers
Source: Sci Rep. 2018 Aug 30;8:13106. doi: 10.1038/s41598-018-30989-3 (PMC6117344; doi:10.1038/s41598-018-30989-3)
Supplement: Supplementary file 1 — Supplementary Dataset [file 41598_2018_30989_MOESM1_ESM.docx]

**OncomiR-10b hijacks the small molecule inhibitor Linifanib in human cancers**

*Paloma del C. Monroig-Bosque, Maitri Y. Shah, Xiao Fu, Enrique Fuentes-Mattei,*

*Hui Ling, Cristina Ivan, Nazila Nouraee, Beibei Huang, Lu Chen, Valentina Pileczki,*

*Roxana S. Redis, Eun-Jung Jung, Xinna Zhang, Michael Lehrer, Rahul Nagvekar,*

*Ana Carolina P Mafra, Maria del Mar Monroig-Bosque, Alexandra Irimie, Carlos Rivera,*

*Calin Dan Dumitru, Ioana Berindan-Neagoe,*

*Edward P. Nikonowicz, Shuxing Zhang, George A. Calin*

**Supplementary Material**

**Supplementary Table 1: Characteristics of breast cancer cell lines used for this study**

| **Cell name** | **Tissue** | **Receptor status** | **Subtype** | **Ki-67** | **p53** | **PD-L1 level** |
| --- | --- | --- | --- | --- | --- | --- |
| MCF-7 | mammary gland, breast; derived from metastatic site: pleural effusion | ER+, PR+, HER2- | Luminal A | Low | WT | Low |
| T47D | mammary gland; derived from metastatic site: pleural effusion | ER+, PR+, HER2- | Luminal A | Low | point mut | Low |
| MDA-MB-231 | mammary gland/breast; derived from metastatic site: pleural effusion | ER-, PR-, HER2- | TNBC | Low | point mut | High |
| MDA-MB-468 | mammary gland/breast; derived from metastatic site: pleural effusion | ER-, PR-, HER2- | TNBC | High | point mut | High |
| HepG2 | hepatocellular carcinoma | Insulin; Insulin-like growth factor II | N/A | N/A | WT | Low |

**Supplementary Table 2: Primers used in this research project**

| **Primer name** | **Type** | **Sequence** | **Experiment** |
| --- | --- | --- | --- |
| pre-miR-10b | Forward | CCCTGTAGAACCGAATTTGTG | RT-PCR of premiR sequence |
| pre-miR-10b | Reverse | TGAAGTTTTTGCATCGACCA | RT-PCR of premiR sequence |
| U6-normalizer | Forward | CTCGCTTCGGCAGCACA | RT-PCR reference gene (control) |
| U6-normalizer | Reverse | AACGCTTCACGAATTTGCGT | RT-PCR reference gene (control) |
| pre-miR-10b | Forward | CGGGATCCTCCTTGGGATGGA | Clone-premiR-DNA coding region |
| pre-miR-10b | Reverse | CGGGATCCAGGAAAAGCTGCT | Clone-premiR-DNA coding region |

**Supplementary Table 3: Antibodies used in this research project**

| **Antibody Name** | **Manufacturer** | **Catalogue ID** |
| --- | --- | --- |
| HOXD10 | Abcam | ab90704 |
| PTEN | Santa Cruz | SC9145 |
| Dicer | Cell Signaling Technologies | 5325 |
| Drosha | Cell Signaling Technologies | 3364S |
| Phosphorylated-VEGFR | Cell Signaling Technologies | 2478T |
| VEGFR | Cell Signaling Technologies | 2479S |
| Phosphorylated-PDGFR | Cell Signaling Technologies | 3161S |
| PDGFR | Cell Signaling Technologies | 3169S |
| β-actin | Sigma-Aldrich | A1978 |
